# Interaction between LINC-ROR and Stemness State in Gastric Cancer Cells with *Helicobacter pylori* Infection 

**DOI:** 10.52547/ibj.25.3.157

**Published:** 2021-03-01

**Authors:** Reihaneh Alsadat Mahmoudian, Maryam Lotfi Gharaie, Roya Abbaszadegan, Mohammad Mahdi Forghanifard, Mohammad Reza Abbaszadegan

**Affiliations:** 1 Immunology Research Center, Mashhad University of Medical Sciences, Mashhad, Iran;; 2 Division of Physiology, Department of Basic Science, Faculty of Veterinary Medicine, Ferdowsi University of Mashhad, Mashhad, Iran;; 3 Department of Biology, Damghan Branch, Islamic Azad University, Damghan, Iran;; 4 Medical Genetics Research Center, Medical School, Mashhad University of Medical Sciences, Mashhad, Iran

**Keywords:** LINC-ROR, Non-coding RNA, SALL4

## Abstract

**Background:**

LINC-ROR, as a cancer-related lncRNA, has vital roles in stem cell survival, pluripotency, differentiation, and self-renewal in hESCs. However, cancer-related molecular mechanisms, its functional roles, and clinical value of LINC-ROR in GC remain unclear. In this study, we aimed to investigate probable interplay between *LINC-ROR* with *SALL4 *stemness regulator and their role with the development of the disease.

**Methods:**

The mRNA expression profile of *LINC-ROR* and* SALL4* was assessed in tumoral and adjacent non-cancerous tissues of GC patients, using quantitative real-time PCR.

**Results:**

Significant *LINC-ROR* underexpression and *SALL4 *overexpression were observed in 55.81% and 75.58% (*p *< 0.0001) of samples, respectively. The expression of *LINC-ROR *and *SALL4* were significantly correlated with each other (*p *= 0.044). There was an association between the underexpression of *LINC-ROR *and sex, stage of tumor progression, tumor type, and location of tumor (*p* < 0.05), and *H.* *pylori *infection with *SALL4* expression (*p* = 0.036). There were also significant correlations between concomitant mRNA expression of *SALL4* and *LINC-ROR* in tumors located at distal noncardiac, positive for *H.* *pylori *infection, tumors with invasion into the muscle layer of the stomach, and grade II tumor (*p* < 0.05).

**Conclusion:**

The clinical results of the *SALL4-LINC-ROR* association propose a probable functional interaction between these markers in tumor maintenance and aggressiveness. Our study can help to understand one of the mechanisms involved in the progression of GC through the function of these regulators.

## INTRODUCTION

Gastric cancer results from interactions between the molecular pathways, genetic/epigenetics alterations, and environmental factors^[^^1^^,^^2^^]^. A series of genetic susceptibilities have been supposed to stem cdebe involved in GC development. These include various polymorphisms as well as alteration in gene expression profile of such as tumor suppressors, inflammation related genes, cellular metabolism genes, EMT markers, transmembrane proteins, and matrix metalloproteinases^[^^3^^]^. Moreover, epigenetic changes can cause the dysregulation of tumor-related markers, leading to GC initiation and progression^[^^4^^]^. Among fundamental epigenetic changes in GC, methylation of CpG islands within the promoter region of certain genes, hydroxymethylation, chromatin remodeling, histone modification, and dysregulation of ncRNAs have been reported^[^^5^^]^.

Evidence has shown a complicated association between ncRNAs and coding genes in the development of human cancers through the regulation of cellular and molecular processes^[^^6^^]^. These ncRNAs are mainly classified into several types of snRNAs, miRNAs, siRNAs, snRNAs, lncRNAs, and lincRNAs^[^^6^^]^. lncRNAs, as transcripts longer than 200 nucleotides, are involved in various biological processes, such as maintenance and modification of chromatin, genome imprinting, DNA methylation, dosage compensation, transcription, splicing, and translation^[^^5^^]^. LncRNAs regulate the expression of their target genes at three prominent levels of transcriptional, post-transcriptional, and epigenetic^[^^7^^]^. Besides, the defective function of lncRNAs can lead to apoptosis, invasion, progression, and metastasis in different human cancers^[^^8^^]^. As a result, lncRNAs are applied as diagnostic, prognostic and therapeutic markers in numerous types of solid tumors such as gastrointestinal cancers^[^^9^^-^^11^^]^. *ANRIL*,* FENDRR*,* AF147447*,* CAT1*,* GAS5*,* HULC*,* MEG3*,* HOTAIR*, *H19*,* GHET1*,* GAPLINC*, and *MALAT1* are among dysregulated lncRNAs recognized in GC^[^^11^^]^. LincRNAs as a member of lncRNAs family demonstrate a highly specific expression pattern in cell or tissue levels ^[^^12^^]^. Moreover, they can often be expressed with their neighboring coding genes and regulate gene expression via affecting nuclear bodies and chromatin complexes^[^^13^^,^^14^^]^. LincRNAs have crucial roles in diverse cellular and molecular processes, e.g. functioning as competing endogenous RNA in modulation of miRNAs expression with the transcriptional network in hESCs, reprogramming somatic cells toward iPSCs, early development in ESCs, and response to cellular stress by coordinating with p53^[^^14^^]^. 

LINC-ROR, a type of lincRNAs with 2.6 kb in length, has broad roles in stress response regulation, coordination in cell-cell and cell-environment interactions, inhibition of miRNAs function, tumor development, and prevention of p53 translation in DNA damage response, to boost pluripotency and stem cell survival^[^^15^^,^^16^^]^. Additionally, LINC-ROR has functional roles in differentiation and self-renewal of hESCs, iPSCs, and ESCs via the regulation of *OCT4*, *SOX2*, and *NANOG* expression^[^^14^^,^^17^^]^. Of note, SALL4, a C2H2 zinc finger TF, correlates with ESC markers of SOX2, OCT4, NANOG, and signaling pathways of BMI-1 and Wnt/β-catenin, leading to control the tumor cell renewal and preserve the pluripotency of ESCs and iPSCs^[^^18^^,^^19^^]^. SALL4 acts as a stemness state regulator in various cancers via ESCC, GC, lung, breast, colon, and hematopoietic cancers^[^^20^^-^^25^^]^. High expression of *SALL4* is correlated with the early steps of tumor development, metastasis to lymph nodes, poor prognosis, and invasion in various malignancies^[^^21^^]^. Studies have investigated the dysregulation of *LINC-ROR* in tumorigenesis by evaluating the increased *LINC-ROR* expression in numerous malignancies, including pancreatic, colon, lung, bladder, endometrial, breast, hepatocellular, nasopharyngeal cancers, and ESCC, as well as its heterogenic function in glioblastoma^[^^15^^,^^26^^-^^28^^]^. Overexpression of *LINC-ROR* was significantly related to the advanced stages of malignancies, metastasis to lymph nodes, and vascular invasion, suggesting that *LINC-ROR* could serve as a prognostic marker in some human cancers^[^^16^^]^. 

Considering the prognostic role of *LINC-ROR*, its function in tumor progression, and its correlation with stemness markers, a probable correlation may be existed between the expression of *LINC-ROR* and *SALL4* in tumorigenesis. Therefore, we aimed in the current study to evaluate the expression of *LINC-ROR* in tumor and adjacent non-cancerous tissues of GC patients and to investigate its probable linkage with *SALL4 *stemness regulator, as well as their correlation with clinicopathological features of patients. 

## MATERIALS AND METHODS

**Patients and tissue samples**

The tumor and adjacent non-cancerous tissues were collected from 86 GC patients who underwent gastrectomy at two affiliated Hospitals of Mashhad University of Medical Sciences (MUMS), Mashhad, Iran, Imam Reza and Omid, from 2008 to 2012. The fresh specimens were quickly transferred to the RNAlater solution (Qiagen, Hilden, Germany) and stored at -20 °C until further use. The information of tumor tissues and patients were characterized according to Union International Cancer TNM classification by a pathologist^[^^29^^]^. Inclusion criteria included patients who had not been received preoperative chemo-radio treatment before the surgery. Based on hematoxylin and eosin analysis, histopathologic examination was performed for all the samples to verify that all cancer cells have at least 70% tumor cells.

**RNA isolation and cDNA synthesis**

Total RNA was extracted from adjacent non-cancerous and tumoral tissue samples using the RNeasy Mini Kit (Qiagen). Five to seven tissue sections (100 mg) were first lysed by adding 1 mL of lysis buffer and then centrifuged at 50,000 ×g for three minutes. All steps were performed as per the manufacturer's instructions. The quantity and purity of total RNAs were measured by a NanoDrop spectrophotometer (WPA, Biowave II^+^, Germany); pure RNA had an A260/A280 absorbance ratio of 1.8 to 2.0. The integrity of RNA preparation was evaluated by the electrophoresis on 1% agarose gel and viewed as the 28S and 18S rRNA bands. Then total RNA was treated with DNase I (Thermo Fisher Scientific, Waltham, MA) in accordance with the manufacturer’s procedures for DNA contamination prevention. The first strand cDNA synthesis was carried out by the primeScript^TM^ RT Reagent kit (Takara, Japan) with 1 µg of treated RNA, oligo(dT), and random primers according to the manufacturer’s instructions (37 °C for 15 min and 85 °C for 5 s). Afterward, the quality of cDNA was verified by the amplification of GAPDH as the control, and cDNA was stored at -20 °C until the real-time PCR.

**Comparative** **real-time PCR analysis**

Comparative real-time PCR experiment was performed based on the Minimum Information for Publication of Quantitative Real-Time PCR Experiments guidelines^[^^30^^]^. We applied the adjacent tissue as a reference sample and compared mRNA expression in tumor with that of adjacent non-cancerous tissues through comparative relative real-time PCR. GAPDH was employed as an internal control to normalize the data in a comparative relative real-time PCR (SYBR Green, AMPLIQON, Denmark) on a LightCycler^®^ 96 Real-Time PCR System thermocycler (Roche, Germany) by gaining the relative Ct values and calculating *LINC-ROR* and *SALL4* mRNA expression through the 2^-ΔΔCt^ method^[^^26^^,^^31^^]^. Tumor mRNA expression higher or less than onefold relative to corresponding gene expression in adjacent non-cancerous tissues was considered as overexpression or underexpression, respectively, whereas the fold changes between -1 and +1 were regarded as no change in the gene expression. All RT-PCRs were performed in duplicates. The thermal cycling conditions, and the sequences of the specific primers set for *LINC-ROR *and *SALL4* are illustrated in Table 1^[^^21^^,^^26^^]^.

**DNA extraction and detection of** ***H*** ***.*** ***Pylori* infection**

 Total genomic DNA was extracted from normal and tumoral tissue samples by enzymatic method (digestion buffer, proteinase K, and RNase) as previously described^[^^32^^]^. Briefly, tissues were lysed in 200 μl of digestion buffer, 20 μl of proteinase K, and 5 μl of RNase at 55 °C for 3 h incubation. Thereafter, 200 μl of phenol/chloroform solution was added to the mixture, and samples were centrifuged at room temperature for one minutes. After the addition of two volumes of absolute ethanol and 0.1 volume of 3 M sodium acetate to the supernatant, the mixture was incubated at -20 °C overnight. The mixture was then centrifuged for 10 minutes, and the DNA pellet was washed with 75% ethanol and resuspended in Tris-EDTA buffer. PCR was accomplished on extracted DNA using specific primers set for *H.* *pylori* genes of *UreC* (*glmM*), *16S rRNA*, and virulence factor of *CagA*. The primer sequences and the thermalprofile for PCR amplification are demonstrated in Table 1. 

**Statistical analysis**

Statistical analyses were performed using SPSS 19.9 software (SPSS, Chicago, IL) and GraphPad Prism 5.0 (La Jolla, CA, USA). The Kolmogorov–Smirnov (K-S) test was applied for normal or non-normal distribution of the data. The correlation between expression of *LINC-ROR* and* SALL4* with different clinicopathological characteristics of GC patients was evaluated by the χ2 or Fisher exact test, paired-samples T-test, independent-samples T-test, and ANOVA. The probable correlation between the *LINC-ROR* and* SALL4 *expression was assessed by Pearson correlation. A *p *< 0.05 was considered a statistically significant level.

**Table 1:** Primer sequences and the thermal cycling used in real-time PCR

**Transcripts**	**Sequence**	**Thermal profile**
*LINC-ROR*	F: ACAAGGAGGAAAGGGCTGAC R: TTCTGGAAGCTAAGTGCACATG	95 °C (15 min) [95 °C (15 s)/63 °C (20 s)/72 °C (20 s)] 40
*SALL4*	F: CCAAAGGCAACTTAAAGGTTCAC R: GAGATCTCATTGGTCTTCACGG	95 °C (10 min) [95 °C (30 s)/58 °C (15 s)/72 °C (30 s)] 40
*16S rRNA*	F:GCTATGACGGGTATCC R:GATTTTACCCCTACACCA	95 °C (5 min) [92 °C (30 s)/55 °C (40 s)/72 °C (40 s)] 40
*UreC (glmM)*	F:AGCTTTTAGGGGTGTTAGGGGTTT R:AAGCTTACTTTCTAACACTAACGC	95 °C (5 min) [92 °C (30 s)/55 °C (40 s)/72 °C (40 s)] 40
*CagA*	F:GATAACAGGCAAGCTTTTGAGG F:CTGCAAAAGATTGTTTGGCAGA	95 °C (5 min) [92 °C (30 s)/55 °C (40 s)/72 °C (40 s)] 40
*GAPDH*	F: GGAAGGTGAAGGTCGGAGTCA R: GTCATTGATGGCAACAATATCCACT	95 °C (10 min) [95 °C (30 s)/58 °C (30 s)/72 °C (30 s)] 40

**Ethical statement**

The above-mentioned sampling protocol was approved by the Ethics Committee of MUMS, Mashhad, Iran (ethical code: 921706). Prior to participation, the informed constant was obtained from all patients participating in this study.

## RESULTS

**Patients’ histopathological characteristics**

The clinicopathological characteristics of 86 GC patients involving 63 males and 23 females enrolled in the present study are summarized in Table 2. The general mean age ± SD of patients at the time of diagnosis was 63.03 ± 10.98 years, and the mean size ± SD of tumor samples was 6.46 ± 3.11 cm. Most cases (64/86, 74.5%) had T3/T4 tumor depth of invasion, 65 out of 86 (75.5%) samples were in stages II/III of tumor, and 73/86 (84.9%) of tumor samples had lymph node metastasis. Based on the histopathological analysis, 60 (69.8%) tumor samples were categorized as intestinal type, whereas 21 (24.4%) and 5 (5.8%) tumors were diffused and mixed types, respectively. Tumors located in the gastric cardia were 37/86 (43%), while 49/86 (57%) was found in noncardia regions of the stomach. The gastric tissue samples were investigated for *H. pylori* infection using PCR for three genes of *16s rRNA*, *UreC*, and *CagA*. Also, 43 (50%) tumor tissue samples were positive for *16s rRNA/UreC*, while 44.2% was positive for *CagA*. 

**Expression analysis of** ***LINC-ROR*** **and** ***SALL4***

**Fig. 1:**
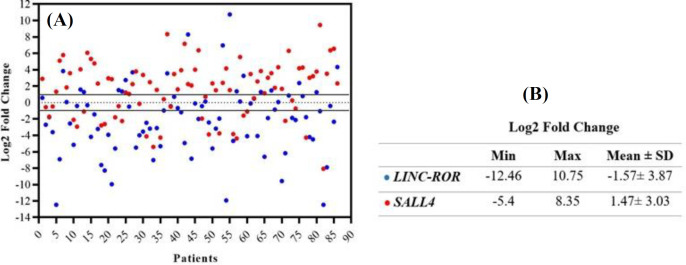
(A) Scatter plot representing descriptive analysis of relative gene expression of *LINC-ROR *and *SALL4* in GC patients. The black lines indicate the thresholds for the over- and under-expression. The range between over- and under-expression shows the cases with normal *LINC-ROR *and *SALL4* mRNA expression; (B) Minimum, maximum, and mean of log2 fold change for the *LINC-ROR* and *SALL4* mRNA expression

**Fig. 2:**
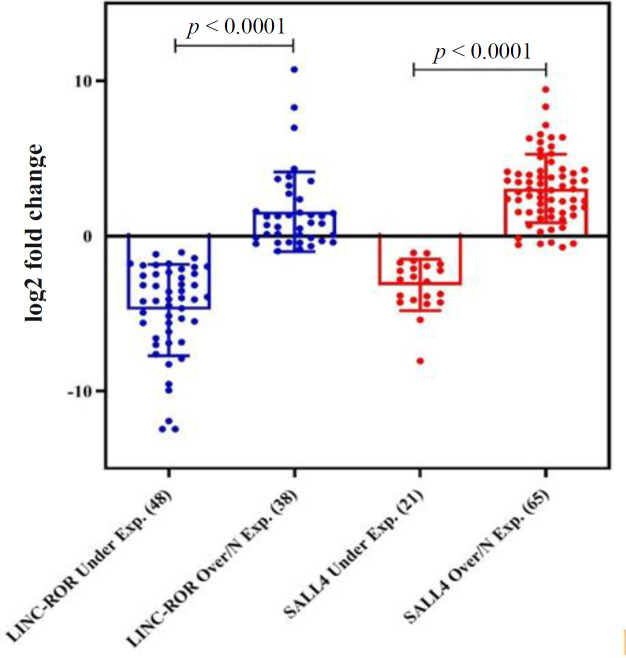
Dot plot representative of relative mRNA expression of *LINC-ROR *and* SALL4* in GC patients. Dot plots represent the lowest, lower quartile, median, upper quartile, and highest observations of fold changes in patients with normal/ over- or under-expressed *LINC-ROR *and *SALL4*

**Fig. 3:**
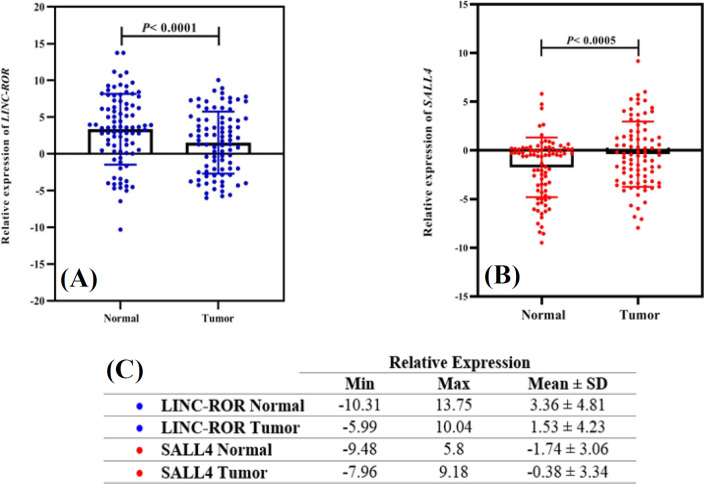
(A and B) The expression alteration of *LINC-ROR *and *SALL4* in GC tissues and adjacent noncancerous tissues. *LINC-ROR *and *SALL4* expression were assessed by qRT-PCR in tissue. Data was evaluated statistically using the two-way ANOVA. (C) Minimum, maximum, and mean of level relative expression for the *LINC-ROR* and *SALL4* in GC tissues and adjacent noncancerous tissues

Relative comparative qRT-PCR indicated that the log2 fold change of *LINC-ROR *and* SALL4* decreased in 55.81% (48/86) and 24.41% (21/86) of tumor samples compared to the adjacent non-cancerous tissues (*p *< 0.0001); however, 38 (44.18%) and 65 (75.58%) samples showed the normal or overexpression of *LINC-ROR* and* SALL4 *(*P *< 0.0001), respectively (Table 4). The profile of mRNA expression in all patients is displayed in Figure 1A as a scatter plot. The minimum and maximum log2 fold changes ranged from -12.46 to 10.57 for *LINC-ROR *and -5.4 to 8.35 for* SALL4*, respectively (Fig. 1B). Expression levels of *LINC-ROR *and *SALL4 *in underexpressed vs. overexpressed or normal expression of tumor specimens are represented in Figure 2 as a dot plot. The mean log2 fold change of *LINC-ROR *and* SALL4* expression level were -1.57 ± 3.87 and 1.47 ± 3.03 (Fig. 1B), respectively. We identified that the *LINC-ROR* level was significantly lower in cancerous tissues compared with the adjacent non-cancerous tissues (normal; Fig. 3A; *p* < 0.0001). Moreover, the SALL4 level was significantly higher in cancerous tissues compared with the paired non-neoplastic gastric tissues (normal; Fig. 3B; *p* < 0.0005). In addition, the average *LINC-ROR *and* SALL4* expression levels of GC tissues were 1.53 ± 4.23 and -0.38 ± 3.34, as well as that of matched adjacent non-cancerous tissues were 3.36 ± 4.81 and -1.74 ± 3.06, respectively, as demonstrated in Figure 3C. We identified that the *LINC-ROR *level was significantly lower in cancerous tissues compared with the adjacent non-cancerous tissues (normal; Fig. 3A; *p *< 0.0001). Moreover, the* SALL4 *level was significantly higher in cancerous tissues compared with the paired non-neoplastic gastric tissues (normal; Fig. 3B; *p *< 0.0005). 

**Table 2:** Clinicopathological features of the 86 GC patients under study

**Factor**	**Patients (%)**
Age (mean ± SD)	63.03 ± 10.98 years
Sex	
Male Female	63 (73.3) 23 (26.7)
Tumor size (mean ± SD)	6.46 ± 3.11 cm
Differentiation	
PD MD WD	20 (23.3) 55 (64) 11 (12.8)
Lymph node metastasis (N)	
N0 N1 N2 N3	13 (15.1) 40 (46.5) 26 (30.2) 7 (8.1)
Grade	
I II III	13 (15.1) 53 (61.6) 20 (23.3)
Stage of tumor progression	
I II III IV	7 (8.1) 15 (17.4) 50 (58.1) 14 (16.3)
Depth of tumor invasion (T)	
T2 T3 T4	22 (25.6) 47 (54.7) 17 (19.8)
Tumor type	
Intestinal Diffused Mixed	60 (69.8) 21 (24.4) 5 (5.8)
Location	
Cardiac Noncardiac	37 (43) 49 (57)
*H. pylori* (*16s rRNA/UreC*)	
Positive Negative	43 (50) 43 (50)
*H. pylori* (*CagA*)	
Positive Negative	38 (44.2) 48 (55.8)

PD, poorly differentiated; MD, moderately differentiated; WD, well differentiated; N0, no. of regional lymph node metastasis; N1, metastasis in one to two regional lymph nodes; N2, metastasis in three to six regional lymph nodes; N3, metastasis in seven or more regional lymph nodes

**Association between concomitant expression of** ***LINC-ROR*** **and** ***SALL4*** **in GC**

**Fig. 4:**
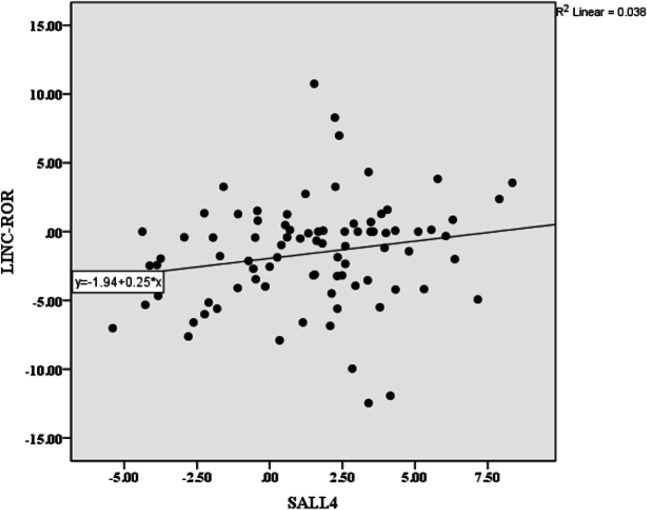
Regression plot illustrating a correlation between the level of *LINC-ROR *and *SALL4* expression (*p* = 0.044)

The mRNA levels of *LINC-ROR *and *SALL4* were significantly correlated with each other (*p *= 0.044; Table 3). Interestingly, the expression level of *LINC-ROR* decreased in tumor specimens with a low level of *SALL4* expression (*p *< 0.05) compared to *SALL4* overexpression samples (*p *= 0.92). Correlation between *LINC-ROR* and *SALL4* expression levels is represented as a regression plot in Figure 4. There was also no significant association between *LINC-ROR* and *SALL4* under- or overexpression (Table 4). 

**Clinicopathological relevance of** ***LINC-ROR*** **and** ***SALL4*** **expression in GC**

To indicate *LINC-ROR *expression on GC progression, we investigated the correlation of *LINC-ROR* expression with patients’ clinicopathological traits (Table 4). Based on the Table, there were some significant correlations between the data, including the stage of tumor progression (*p *= 0.029), the tumor type (*p *= 0.05), and the location of tumor (*p *= 0.048) with

*LINC-ROR *mRNA expression. Among samples with *LINC-ROR* underexpression, 16.2% (14/86) and 29% (25/86) samples were located in cardiac and noncardiac regions, and 31.3% (27/86) indicated intestinal tumor type compared with diffused or mixed type 16.1% (14/86) GC. Moreover, there was a correlation between sex and *LINC-ROR *expression (*p *= 0.05), but correlation between *LINC-ROR* and other clinicopathological factors was insignificant. However, GC patients with underexpression of *LINC-ROR *were prone to N1/N2 steps of lymph node metastasis (47.1%), indicating that metastasis of tumor cells to the lymph node was declined via decreasing in *LINC-ROR *expression. Most specimens with *LINC-ROR* underexpression had grade II (29%) and 13.9% was invaded to the adventitia (T3). There was not any significant correlation between the level of *SALL4* mRNA expression with patients’ clinicopathological traits (Table 4). 

**Correlation between** ***LINC-ROR*** **and** ***SALL4*** **expression and** ***H. pylori*** **infection**

A significant correlation was found between *SALL4* mRNA expression and *H. pylori* infection in which 50% (43/86) and 40.7% (35/86) gastric tumor samples were positive for *16s rRNA/UreC* (*p *= 0.047) and *CagA *(*p *= 0.036), respectively (Table 4). Furthermore, our results indicated that no significant correlation between *LINC-ROR* expression and *H. pylori* infection in GC. The majority of *LINC-ROR* underexpression samples were positive for *16s rRNA/UreC* (48.7%) and *CagA* (64.1%). 

**Table 3:** Association between *LINC-ROR *and* SALL4* expression in GC samples

	***SALL4***	***SALL4 *** **underexpression**	***SALL4*** **overexpression**
*LINC-ROR* Pearson correlation Sig. (two‐tailed)	0.218* 0.044		
*LINC-ROR *underexpression (N) Pearson correlation Sig. (two‐tailed)		11 0.043 0.900	16 0.143 0.597
*LINC-ROR *overexpression (N) Pearson correlation Sig. (two‐tailed)		0	7 -0.578 0.174

*Correlation is significant at the 0.05 level

**Correlation between the concomitant expression of** ***LINC-ROR*** **and** ***SALL4*** **with different pathological variables**

There was significant correlation of the concomitant expression level of* LINC-ROR* with *SALL4* in specimens positive for *H.* *pylori *infection (*p *= 0.03). The co-expression of *LINC-ROR* and *SALL4* was significantly associated with the depth of tumor invasion (T2; *p *= 0.002) and tumor grade of II (*p *= 0.05). Moreover, co-expression of *LINC-ROR* and *SALL4* in tumor samples was significantly correlated with moderately differentiated (*p *= 0.05). Furthermore, co-expression of both *LINC-ROR* and *SALL4* in the tumors located at distal noncardiac region (*p *= 0.05). We found no significant correlation between co-expression of *LINC-ROR* and *SALL4* with metastasis of tumor cells into lymph nodes, stage of tumor progression, and tumor type. The correlations between the co-expression of *LINC-ROR* and *SALL4* with different pathological states of the patients are summarized in Table 4. 

## DISCUSSION

Recent studies in the last decade have displayed high incidence and poor diagnosis with a survival rate of 31% in GC patients^[^^33^^,^^34^^]^. Identification of new biomarkers is critical in early detection, diagnosis, and treatment that would help to improve the quality of life in cancer patients^[^^35^^]^. Recent data have illustrated that lncRNAs have widely participated in biological procedures, and their misexpression has an impact on the pivotal function of molecules involved in signaling pathways and tumor pathogenesis^[^^28^^]^. 

LncRNAs play key roles in tumorigenesis and many aspects of cancer development; therefore, evaluating the probable correlation between *LINC-ROR *and* SALL4 *in GC development is needed. We reported for the first time the significantly declined expression of *LINC-ROR *at the transcript level in GC tissues. Moreover, we indicated a significant correlation between concomitant expression of *LINC-ROR* and stemness transcriptional factor *SALL4* with clinicopathological features, including *H. pylori *infection, depth of tumor invasion, tumor location, differentiation, tumor grade, and sex in gastric tissues. These data display that co-expression of *LINC-ROR *and* SALL4 *may contribute to the development and progression of GC.

LincRNA function as TSG or oncogene in pathogenesis and development of different cancers suggests that they take a part in various cellular and molecular processes, from ESC commitment to tumorigenesis-associated gene expression system as vital regulators^[^^36^^]^. Interaction of lincRNAs with other coding genes and ncRNAs can change various mechanisms, e.g. chromatin modifications, transcription, and post-transcription processes, acting as protein and RNA decoys in leukemia and solid malignancies^[^^37^^]^. Some lncRNAs are identified as biomarkers in the diagnosis and prognosis of malignancies, including H19, HOTAIR, and MALAT1^[^^38^^]^. Moreover, aberrant expression of LINC-ROR, located at 18q21, has been indicated in the progression of some human malignancies^[^^36^^]^. The expression level of *LINC-ROR* was significantly associated with metastasis to lymph nodes, vascular invasion, and advanced stages of tumor progression, where its expression activated during tumor progression^[^^16^^]^. It has been shown that dysregulation of *LINC-ROR* is correlated with poor prognosis and overall shorter survival in GC patients, implying that LINC-ROR may be identified as an independent prognostic marker and a vital modulator for GC^[^^31^^]^. Moreover, increased* LINC-ROR* expression in CD133^+ ^GC stem cells was correlated with proliferation, invasion, and apoptosis inhibition, as well as with the overexpression of *OCT4*, *SOX2*, and *NANOG*^[^^39^^]^. Interestingly, we found *LINC-ROR* downregulation in GC tissues, and this observation is not in accordance with those reported in a previous study^[^^40^^]^. Therefore, we can conclude that *LINC-ROR* demonstrates a heterogeneous expression level in different populations of GC patients. In this regard, a former study has examined the expression of *LINC-ROR *in various cell lines and human tumor tissue samples and reported that the expression of *LINC-ROR* increases in cervical, esophageal, and ovarian cancers, but decreases in breast, colon, sarcoma and melanoma cancers. In addition, underexpression of *LINC-ROR* has been observed in somatic cancer cell lines, except for undifferentiated ESCs and embryonic carcinoma cell lines^[^^40^^]^. It is noteworthy that *LINC-ROR* has also been indicated heterogeneous expression levels in GC. Our outcomes propel the hypothesis that the underexpression of *LINC-ROR* in GC tissues may be due to more prevalent of monosomy chromosome 18 (location of *LINC-ROR*) compared with trisomy 18 (based on the Mitelman database of chromosome aberrations and gene fusions in cancer^[^^39^^,^^41^^]^. Another hypothesis about the diversity of *LINC-ROR* expression in GC is that different small subpopulations from the cancerous mass of GC stem cells may lead to the heterogeneous expression of *LINC-ROR* in different populations^[^^40^^]^. Our results indicated that the underexpression of *LINC-ROR* is associated with tumor type and location, stage of tumor progression, and gender of patients. In line with our reports, the heterogeneous expression level of *LINC-ROR* has been illustrated in glioblastoma, as the up-regulation of *LINC-ROR* was related to overall survival and poor progression^[^^42^^]^. On the other hand, the underexpression of *LINC-ROR* is correlated with the mRNA expression levels of *SOX11* and *KLF4 *in glioma^[^^36^^]^. Herein, we have sought to examine the significant changes in the expression level of *LINC-ROR* in some malignancies. It has been illustrated that the upregulation of* LINC-ROR* is associated with cell proliferation, EMT, migration, invasion, metastasis, poor prognosis, inhibition of *NANOG* expression, and alteration of cancer stem-like cells properties via the regulation of miRNAs in pancreatic cancer^[^^43^^,^^44^^]^. Recent studies have found that *LINC-ROR* overexpression can trigger proliferation, EMT, progression, and metastasis via interaction with miRNAs and the TGF-β signaling pathway in breast cancer^[^^45^^,^^46^^]^. Moreover, *LINC-ROR *silencing results in the inhibition of cell proliferation, invasion, and EMT through reducing *CDH1* expression and increasing the expression of *ZEB1/2* and *Vimentin *in ESCC tumor tissues^[^^26^^,^^47^^]^. Increased *LINC-ROR *expression was found in bladder cancer tissues and cell lines, leading to the induction of EMT, promotion of cell proliferation, migration, invasion, and tumor progression^[^^28^^]^. It has been divulged that *LINC-ROR* is overexpressed and linked to the hypoxia network via modulating miR-145-HIF-1α expression in hepatocellular cancer^[^^48^^]^. *LINC-ROR *is upregulated in lung cancer stem cell and non-small cell lung cancer and associated with poor prognosis^[^^27^^]^. Evidence has shown that LINC-ROR, as an epigenetic regulator implicated in lineage commitment, sorely associates with tumorigenesis and stemness^[^^49^^]^. 

**Table 4:** Expression profile of *LINC-ROR* and *SALL4* in different clinicopathological features of the enrolled GC patients

**Factor**	***LINC-ROR***		***p*** ***value***	***SALL4***		***p value***	**Coexpression of markers** **(** ***p value*** **)**
	**─ / ** **↑** ** (%)**	**↓ (%)**		**─ / ** **↑** ** (%)**	**↓ (%)**		
Sex Male Female	26 (30.2) 13 (15.1)	37 (43) 10 (11.6)	**0.05** ^*^	25 (29) 12 (13.9)	38 (44.1) 11 (12.7)	NS	0.246 0.031^*^
Differentiation							
PD MD WD	12 (13.9) 30 (34.8) 5 (5.8)	8 (9.3) 25 (29) 6 (6.9)	NS	10 (11.6) 23 (26.7) 4 (4.6)	10 (11.6) 32 (37.2) 7 (8.1)	NS	**0.07** 0.02^*^ 0.48
Lymph node metastasis (N)							
N0 N1 N2 N3	7 (8.1) 17 (19.7) 11 (12.7) 4 (4.6)	6 (6.9) 23 (26.7) 15 (17.4) 3 (3.4)	NS	7 (8.1) 14 (16.2) 14 (16.2) 2 (2.3)	6 (6.9) 26 (30.2) 12 (13.9) 5 (5.8)	NS	**0.61** **0.19** **0.13** **0.7**
Grade							
I II III	7 (8.1) 28 (32.5) 12 (13.9)	6 (6.9) 25 (29) 8 (9.3)	NS	6 (6.9) 21 (24.4) 10 (11.6)	7 (8.1) 32 (37.2) 10 (11.6)	NS	**0.62** 0.023^*^ 0.081
Stage of tumor progression							
I II III IV	4 (4.6) 5 (5.8) 33 (38.3) 5 (5.8)	3 (3.4) 10 (11.6) 17 (19.7) 9 (10.4)	**0.029** ^*^	5 (5.8) 5 (5.8) 22 (25.5) 5 (5.8)	2 (2.3) 10 (11.6) 28 (32.55) 9 (10.4)	NS	**0.16** **0.23** **0.34** **0.59**
Depth of tumor invasion (T)							
T2 T3 T4	11 (12.7) 26 (30.2) 10 (11.6)	11(12.7) 10 (11.6) 2 (2.3)	NS	9 (10.4) 20 (23.25) 8 (9.3)	13 (15.11) 27 (31.39) 9 (10.4)	NS	**0.002** ^*^ 0.96 0.76
Tumor type							
Intestinal Diffuse Mixed	33 (38.3) 11 (12.7) 3 (3.4)	27 (31.3) 11 (12.7) 3 (3.4)	**0.05** ^*^	25 (29) 8 (9.3) 4 (4.6)	35 (40.6) 13 (15.11) 1 (1.1)	NS	0.19 0.14 0.75
Location							
Cardiac Noncardiac	23 (26.7) 24 (27.9)	14 (16.2) 25 (29)	**0.048** ^*^	15 (17.4) 22 (25.5)	22 (25.5) 27 (31.39)	NS	0.77 0.021*
*H.* *pylori* (*16s rRNA/UreC*)							
Positive Negative	23 (26.7) 24 (27.9)	19 (22) 20 (23.2)	NS	24 (27.9) 13 (15.1)	19 (22) 30 (34.8)	**0.047** ^*^	**0.03** ^*^ 0.96
*H.* *pylori* (CagA)							
Positive Negative	23 (26.7) 24 (27.9)	25 (29) 14 (16.2)	NS	12 (13.9) 25 (29)	23 (26.7) 26 (30.2)	**0.036** ^*^	**0.041** ^*^ 0.87

PD, poorly differentiated; MD, moderately differentiated; WD, well differentiated; N0, no. of regional lymph node metastasis; N1, metastasis in one to two regional lymph nodes; N2, metastasis in three to six regional lymph nodes; N3, metastasis in seven or more regional lymph nodes; NS, non-significant correlation

The stemness state is involved in EMT and tumor growth in different steps of tumorigenesis through a set of TFs^[^^21^^]^. It has been demonstrated that *LINC-ROR* regulates the pluripotency levels of TFs, including *SALL4*, *LIN28*, *OCT4*, *SOX2*, and *NANOG* via acting as a miRNA-145 sponge, leading to the preservation of hESCs and cancer stem cells undifferentiated status^[^^14^^,^^50^^,^^51^^]^. Moreover, the dysregulation of *LINC-ROR* is correlated with the upregulation of stemness TFs of *OCT4, SOX2, *and *NANOG* and the pluripotent factor of *CD133* in GC stem cells^[^^39^^]^. The expression of *LINC-ROR* represses ESC differentiation, resulting in boosting the survival of ESCs and iPSCs through suppressing cellular stress pathways^[^^52^^]^. LINC-ROR stimulates cancer stem cell phenotype and ESCC progression through the deregulation of SOX9, as a TF involved in embryogenesis and maintenance of stem/progenitor cells, to coordinately promote cell proliferation, motility, self-renewal capacity, and cancer stemness state^[^^53^^]^. Expression of* LINC-ROR* has been reported to be upregulated in undifferentiated oral squamous cell carcinoma and correlated with the overexpression of *KLF4, C-MYC, OCT4,* and *SOX2*^[^^49^^]^. According to the role of *SALL4* in stemness state of GC cells as well as the function of *LINC-ROR* as a cell stemness regulator, we assessed whether there is a correlation between the expression of *SALL4* and *LINC-ROR* in GC^[^^49^^,^^54^^]^. Accordingly, we indicated a significant co-expression of markers in the GC tissues patients. These tumor tissues are located in noncardiac regions with invasion into the muscle layer of the stomach (T2). In addition, these samples were in grade II tumor and moderate differentiation status, introducing this correlation as an effective axis of markers in GC patients. Our findings revealed that the co-expression of *LINC-ROR *and *SALL4 *was significantly correlated with each other in the early steps of tumor development in patients. Remarkably, the association between *LINC-ROR* and *SALL4* expression may enhance the stemness characteristics and promote the progression of GC. Induction of EMT via the deregulation of* LINC-ROR* may activate the expression of stemness marker, *SALL4*, as a TF with an oncogenic role in GC, to promote cancer phenotype. It has been illustrated that lncRNA differentiation antagonizing non-protein coding RNA boosts the proliferation, metastasis, and invasion in GC cells through activating SALL4, to adjust the expression of EMT regulators, such as *TWIST*, *SLUG*, and *E-cadherin*^[^^19^^]^. However, no reports are available on the clinical importance of LINC-ROR and SALL4 in GC aggressiveness. Our results may provide evidence for the possible linkage between LINC-ROR and SALL4 stemness markers in GC tumorigenesis.

There is a direct interaction between *H. pylori* infection and GC as change in the host cell microenvironment can activate oncogenic pathways^[^^32^^]^. Identification of epigenetic alterations can assist to predict and diagnose cancer patients’ prognosis^[^^55^^]^. Accordingly, the deregulation of lncRNAs in combination with *H.* *pylori* infection can be worthwhile in predicting markers involved in GC development^[^^56^^]^. The *LINC-ROR *expression pattern in gastric cells infected by *H. pylori* has never been reported until now. Considering the widespread functions of lncRNAs in molecular processes and their role in pathogenesis and tumorigenesis, the aberrant expression of *LINC-ROR *may relate to *H.* *pylori* infection in GC patients. Thus, in the present study, we have shown that the significant co-expression of *LINC-ROR *and *SALL4* is correlated with *H. pylori*-positivity. Moreover, the *LINC-ROR* expression declined in specimens with *H. pylori* infection, and dysregulation of *LINC-ROR* may be correlated with the *H. pylori*-related carcinogenesis. Previous studies have indicated that *H.* *pylori* infection could deregulate the downstream target genes of the Wnt signaling pathway, such as *CDX1*. Dysregulation of *CDX1 *can induce the expression level of stemness markers such as *SALL4* to transform gastric epithelial cells into dedifferentiated stem/progenitor-like cells, which then converts to intestinal metaplasia^[^^57^^,^^58^^]^. Moreover, it has been indicated that the infection of* H.* *pylori* alters the expression level of lncRNAs, leading to *H.* *pylori*-related cancer^[^^56^^]^. Our results may consider a signaling axis of *LINC-ROR-SALL4* through its correlation with *H.* *pylori* infection, to display the possible crosstalk between *LINC-ROR *and *SALL4* in GC tumorigenesis. 

 Overall, our findings reveal the downregulation of* LINC-ROR* in GC tissues. The decreased expression of *LINC-ROR* may be affected by mechanism underlying *SALL4* deregulation and *H.* *pylori* infection, which may provide a new viewpoint to understand the dual function of *LINC-ROR* as oncogene or TSG in GC. Our data also suggest a linkage between tumorigenesis and dedifferentiation and stemness status. However, our results propose that concomitant dysregulation of *LINC-ROR* and *SALL4* in transcript level may have a potential role in tumor initiation and invasion of GC. This conclusion requires further functional analysis for better understanding of the mechanisms involved in the pathogenesis and progression of GC.
